# Digital health interventions for all? Examining inclusivity across all stages of the digital health intervention research process

**DOI:** 10.1186/s13063-024-07937-w

**Published:** 2024-01-30

**Authors:** Rebecca A. Krukowski, Kathryn M. Ross, Max J. Western, Rosie Cooper, Heide Busse, Cynthia Forbes, Emmanuel Kuntsche, Anila Allmeta, Anabelle Macedo Silva, Yetunde O. John-Akinola, Laura M. König

**Affiliations:** 1 Department of Public Health Sciences, School of Medicine, University of Virginia, PO Box 800765, Charlottesville, VA 22908-0765 USA; 2 Department of Clinical & Health Psychology, College of Public Health & Health Professions, University of Florida, PO Box 100165, Gainesville, FL 32610-0165 USA; 3 Department for Health, University of Bath, Claverton Down, Bath, BA2 7AY UK; 4 Nuffield Department of Primary Care Health Sciences, University of Oxford, Woodstock Road, Oxford, UK; 5 Leibniz Institute for Prevention Research and Epidemiology- BIPS, Achterstraße 30, 28359 Bremen, Germany; 6 Hull York Medical School, University of Hull, Allam Medical Building, Cottingham Road, Hull, UK; 7 Centre for Alcohol Policy Research, La Trobe University, Plenty Road and Kingsbury Drive, Melbourne, 3086 VIC Australia; 8 University of Bayreuth, Fritz-Hornschuch-Straße 13, 95326 Kulmbach, Germany; 9 Instituto de Estudos Em Saúde Coletiva IESC/ Universidade Federal Do Rio de Janeiro /Leibiniz Science Campus Digital Public Health/Ministério Público Do Estado Do Rio de Janeiro, Rua das Bauhineas 200, Bl B 1602, Península, Barra da Tijuca, Rio de Janeiro, 22776-090 Brazil; 10 Department of Health Promotion and Education, Faculty of Public Health, College of Medicine, University of Ibadan, College of Medicine, Queen Elizabeth Road, UCH Campus, Ibadan, Nigeria; 11 University of Bayreuth, Faculty of Life Sciences: Food, Nutrition and Health University of Vienna, Faculty of Psychology, Wächtergasse 1, 1010 Vienna, Austria

## Abstract

Digital interventions offer many possibilities for improving health, as remote interventions can enhance reach and access to underserved groups of society. However, research evaluating digital health interventions demonstrates that such technologies do not equally benefit all and that some in fact seem to reinforce a “digital health divide.” By better understanding these potential pitfalls, we may contribute to narrowing the digital divide in health promotion. The aim of this article is to highlight and reflect upon study design decisions that might unintentionally enhance inequities across key research stages—recruitment, enrollment, engagement, efficacy/effectiveness, and retention. To address the concerns highlighted, we propose strategies including (1) the standard definition of “effectiveness” should be revised to include a measure of inclusivity; (2) studies should report a broad range of potential inequity indicators of participants recruited, randomized, and retained and should conduct sensitivity analyses examining potential sociodemographic differences for both the effect and engagement of the digital interventions; (3) participants from historically marginalized groups should be involved in the design of study procedures, including those related to recruitment, consent, intervention implementation and engagement, assessment, and retention; (4) eligibility criteria should be minimized and carefully selected and the screening process should be streamlined; (5) preregistration of trials should include recruitment benchmarks for sample diversity and comprehensive lists of sociodemographic characteristics assessed; and (6) studies within trials should be embedded to systematically test recruitment and retention strategies to improve inclusivity. The implementation of these strategies would enhance the ability of digital health trials to recruit, randomize, engage, and retain a broader and more representative population in trials, ultimately minimizing the digital divide and broadly improving population health.

Inequalities in health are widely recognized; an individual’s health status and access to and use of health care services are affected by a host of factors, from individual lifestyle to social and community networks, living and working conditions, and general socio-economic, cultural, and environmental conditions [1]. Although population health aims to narrow these inequalities, they may actually be reinforced through current research practices. For instance, study factors such as how research study information is communicated (e.g., language used) and the requirements for taking part in a study (e.g., time and literacy required), along with individual factors such as perceived benefits and harms as well as trust of both healthcare professionals and the research process impact which populations can and want to take part in research [2, 3] and often limit participation from minoritized groups. Although certain groups (e.g., those with health conditions preventing them from safely engaging in an intervention) may be intentionally excluded, the exclusion of minoritized populations in research evaluating intervention efficacy/effectiveness is frequently unintended. Indeed, if researchers use the “usual” research methods without actively reflecting on the implications of this choice on the conclusions that can be drawn from data collected in this manner [4], this oversight can produce broader harm as evidence for effectiveness may be lacking for populations that may need support the most, such as populations with low socioeconomic status (SES), that consistently experience less favorable health outcomes and higher mortality rates than high SES populations [5, 6]. For example, one review demonstrated that individual-focused interventions such as dietary counseling can widen social inequalities by being less effective in minoritized populations, potentially because they require more individual agency (which minoritized populations tend to have less of due to a host of factors, including structural barriers) [7].

The advancement of digital technologies offers many possibilities for improving health, as remote interaction can enhance reach and access to underserved groups of society [8, 9]. However, research evaluating the reach, engagement, efficacy/effectiveness, and retention of digital health interventions demonstrates that such technologies do not equally benefit all and that some in fact seem to reinforce a digital health divide [10, 11]. This divide is partially related to issues of access to digital technologies, which may be less accessible to marginalized populations (e.g., people living in housing without Internet access or with lower Internet bandwidth, people living with disabilities, or racial and ethnic minorities; see [12] for a discussion). Beyond this, the broader research process can contribute to widening the digital health divide [13]. Thus, the aim of this article is to highlight and reflect upon study design decisions that might unintentionally enhance inequities across key research stages—recruitment (i.e., hearing about the study and initially indicating interest), enrollment (i.e., successfully making it through the screening procedures), engagement (i.e., actively participating in the intervention through behaviors such as attendance at sessions and self-monitoring health behaviors), efficacy or effectiveness (i.e., achieving the desired health improvement in ideal and broader settings), and retention (i.e., remaining involved in the study and participating in all phases of data collection) (Fig. 1). By better understanding these potential pitfalls, we may contribute to narrowing the digital divide in health promotion [13].

**Fig. 1:**
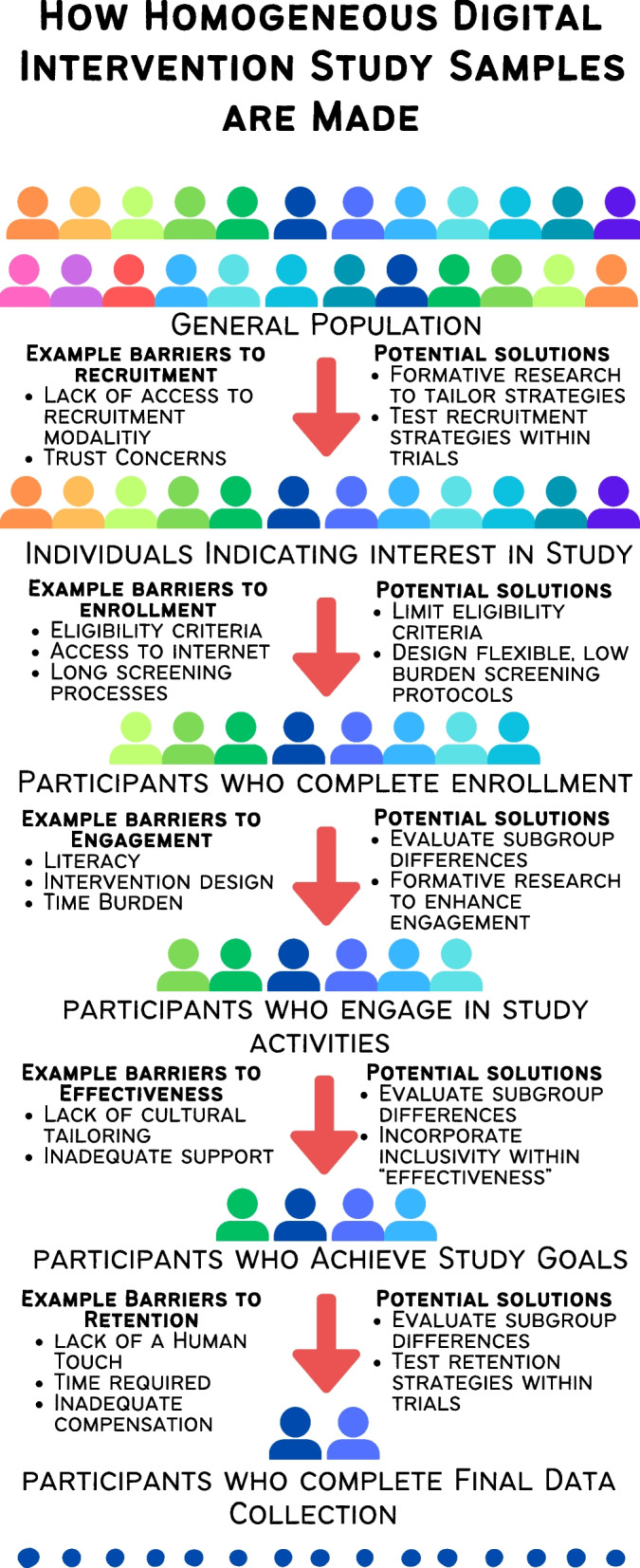
How homogeneous digital intervention study samples are made

## Documenting the digital divide at the different stages of the research process

### Recruitment

In order to accept or refuse participation in research, one has to become aware of existing research studies and be provided with opportunities to take part [14], such as through different means of offline or online recruitment methods that tend to reach different audiences [15]. In addition, there is often an inadequate representation of racial and ethnic minoritized groups in clinical trials, attributed to distrust and fear of research and the medical profession or medical institutions [16] resulting from historic racial injustices [17, 18]. For instance, Pratap et al. compared the proportion of racial and ethnic minoritized participants recruited into large remote digital health trials conducted in the USA to United States Census data, indicating that the proportion of racial and ethnic minoritized participants recruited was substantially lower than would be expected given the states’ population averages [19]; on average, 9.2% fewer participants who identified as African American/Black and 8.1% fewer participants who identified as Hispanic/Latino were recruited into clinical trials compared to Census data.

### Enrollment

Once initial interest to participate in a research study or trial is established, the screening stage follows, wherein specific criteria are applied to each participant to determine eligibility for enrollment into the study. Eligibility criteria might refer to age, gender, or medical history and importantly often include access, previous experience with, or habitual use of digital technologies. Access to, and use of, digital devices and the Internet is often lower in those who are of older age, have lower education status, have lower socioeconomic status, are living in rural areas, or are from minoritized ethnic groups [20, 21]. The perceived ubiquity of Internet-enabled mobile phone ownership is frequently regarded as an opportunity to improve patient engagement in clinical trials of digital health technologies [22]; however, access is not universal, and participation in digital health trials usually requires access to either a smartphone, computer, or tablet along with a reliable Internet connection. Moreover, a recent meta-regression across 80 trials found that 30% of trials involved behavioral “run-ins” (i.e., periods in which potential participants had to demonstrate adherence to specific behavioral tasks) and up to four pre-enrollment steps, likely further limiting participation and adversely impacting generalizability of study findings [23].

### Engagement

Once eligible participants who have access to appropriate devices and an Internet connection enroll in a trial, digital intervention engagement can be suboptimal (i.e., when participants do not use intervention tools at all or as consistently as recommended [11]). This is critical as intervention engagement often directly influences treatment outcomes; for instance, more frequent self-monitoring is associated with greater health behavior change, but certain population sub-groups are less likely to self-monitor [11], which may result in reduced intervention effectiveness [24, 25]. Subpar engagement is often attributed to a low level of digital health literacy (i.e., the competencies, skills, and knowledge required to navigate such technologies [18]); however, digital literacy requirements also depend on the usability and design of digital health technologies [18]. Several strategies such as personalization, gamification, and reminders have been linked to increased engagement [26]. For example, in a teenage pregnancy prevention program, engagement was highest for text messages that included quizzes [27]. In another study, a gamified intervention that included self-chosen, immediate step goals was found to increase physical activity engagement among individuals living in low-income neighborhoods, while other types of step goals within the gamified intervention did not result in consistent increases in physical activity [28]. Yet, other studies have shown that users report some of these strategies to not be helpful or even that they might even put them off using the app altogether [29]. If linked to social inequality indicators, these engagement strategy preferences may risk widening instead of reducing inequalities.

### Efficacy and effectiveness

The aforementioned differences in engagement can translate into differential effects for digital health interventions on desired outcomes, both in tightly controlled efficacy trials and broader, “real world” effectiveness studies. For example, a systematic review found that digital interventions increased physical activity in participants from high-SES backgrounds but no impact was observed in participants from low-SES backgrounds [10]. Similarly, age and gender differences in efficacy and effectiveness have been reported for mobile interventions for the management of weight-related behaviors including diet [11], potentially due to factors such as intervention tailoring. It is likely that the digital divide is not limited to interventions promoting physical health, but also affects the outcomes of mental health interventions. For instance, in a recent study, White women benefited from digital cognitive behavioral therapy to reduce insomnia, while Black women did not [30]. Despite evidence that some digital health interventions produce differential outcomes between populations, reporting of social inequality indicators in relation to the effectiveness of digital mental health interventions is scarce, which complicates drawing conclusions [31]. Aside from differences in engagement, there may be other factors that result in differences in efficacy and effectiveness. For instance, the relationship between intention and behavior is moderated by education [32]; potentially, highly educated individuals might be more aware of how they can reach the desired levels of behavior or are better able to put suggestions into action (e.g., engage in exercise, consume fruits and vegetables).

### Retention

Digital health studies often suffer from high attrition rates [33], greatly affecting external validity and ability to generalize study results to the broader target population. Predictors of dropout include younger age [19, 34–36], lower education levels [34, 36], lower SES [37], lower confidence in the ability to make target health behavior changes [36], and poorer health status at baseline [34]. Previous research indicates that study retention may be overall improved by traditional means (e.g., offering financial incentives [38, 39] and providing reminders of assessment tasks), but there are additional considerations related specifically to digital health research. In particular, the use of live (i.e., “human”) versus automated reminders [34], along with the use of shorter, more frequent assessment tasks versus longer questionnaire batteries [35] and passive measurement (i.e., through smartphone applications or wearable sensors) [40] can reduce participant burden and increase retention in longer-term studies. Moreover, assessment completion can be incentivized by the return of individualized feedback reports based on the data collected [40].

## Implications and recommendations

In this paper, we have summarized inequalities that may unintentionally occur across all phases of the research process (i.e., recruitment, randomization, engagement, efficacy/effectiveness, and retention), that may over-represent the subgroups that may need the digital health interventions the least. There is a clear need to make consistent and concerted efforts to ensure digital health research is more inclusive. For starters, we propose the standard definition of ‘effectiveness’ in digital intervention trials should be revised to include a measure of inclusivity. At a minimum, studies should measure sociodemographic characteristics at first contact to be able to report in detail the representativeness of the participants recruited, randomized, and retained relative to the demographic composition of the target population [41]. The following set of additional recommendations could enhance inclusivity across various stages of digital health intervention studies.

### Recruitment

1. Researchers should conduct formative work to generate ideas for recruitment modalities that will reach diverse population sub-groups.
2. The implementation of recruitment techniques should be paired with continuous data-driven evaluation of the success of each recruitment modality (both overall and for specific minoritized groups). Conducting these evaluations in a systematic way within clinical trials (and reporting outcomes in the literature), similar to the Medical Research Council’s Systematic Techniques for Assisting Recruitment to Trials project [42], can more broadly enhance knowledge of successful recruitment strategies for various sub-groups.

### Enrollment

3. Researchers should select eligibility criteria carefully to avoid selecting criteria that are unnecessarily narrow.
4. Every effort should be made to use flexible screening protocols that focus on decreasing participant burden.
5. When appropriate, researcher should provide necessary technology free of charge to those who do not have it [43].

### Engagement

6. Evaluation of potential subgroup differences in engagement is critical; researchers should conduct sensitivity analyses examining sociodemographic differences in digital intervention engagement during formative, intervention-development periods (i.e., not waiting just to examine differences in efficacy and effectiveness trials).
7. Attempts to establish specific strategies for improving engagement should be made by engaging participants from historically under-represented groups in the design of the intervention [44, 45], using established patient and public involvement techniques to establish challenges and potential solutions (see [46] for a case study).

### Efficacy/effectiveness

8. Similar to the sensitivity analyses suggested previously, researchers should conduct sensitivity analyses examining sociodemographic differences in digital intervention efficacy and effectiveness (preferably pre-planned and powered for in the study design phase, but at the very least conducted as exploratory analyses) to enable scrutiny of how likely the intervention is to benefit those individuals who likely need the support the most. As an example, we present a sensitivity analysis from the Moms Fit 2 Fight trial, which tested a behavioral gestational weight gain intervention with digital components among military personnel and their family members [47]. While the overall effect of this intervention was significant, sensitivity analyses revealed that the intervention effect on gestational weight gain was only significant for women who identified as White; the intervention did not significantly benefit women who identified as Black or with those from other racial groups [48], suggesting a need to adapt the intervention specifically for these populations. If these sensitivity analyses were not conducted, key decision-makers may have not been aware that further consideration of this intervention may be necessary for particular populations.
9. Better transparency of reporting on the inclusivity of key outcomes will facilitate richer evaluation of digital health technologies effectiveness, cost-effectiveness, acceptability, and safety, conforming to Health Technology Assessment frameworks that guide the adoption of new innovations. Indeed, these frameworks such as EUnetHTA core evaluation model [49] place emphasis on the ethical and social aspects of health technologies, and researchers should provide sufficient evidence to inform the wholistic judgement of digital health interventions.

### Retention

10. Although it is common to report sample demographics of study participants at baseline (i.e., describing the sample of individuals recruited and ultimately enrolled), we also recommend that researchers examine sociodemographic characteristics related to retention to detect subgroups that may not be fully represented in the outcome analyses.
11. Studies embedded within larger clinical trials should also be employed to systematically evaluate different retention strategies that may be required for varying subgroups of participants, similar to the methods in the PROMETHEUS program [50].

### Reporting

12. Preregistration of trial protocols should include (1) recruitment benchmarks for sample diversity, (2) comprehensive lists of sociodemographic characteristics assessed, and (3) analysis plans that proactively examine the representativeness of the sample in terms of recruitment, randomization, engagement, efficacy/effectiveness, and retention. Suggestions for an extension of study pre-registration forms have already been proposed for health research more broadly that may serve as a blueprint [51].
13. When reporting sociodemographic characteristics of study samples for digital intervention trials (again not only reporting on participants screened and recruited/randomized but also sociodemographic characteristics related to retention), researchers should consider a wide range of potential inequality indicators beyond the commonly reported age and gender variables. Cochrane’s PROGRESS plus framework provides an overview of socio-demographic factors that have been associated with health disparities [45]. Indeed, several factors listed in this framework have been associated with the digital divide, as evidenced in the meta-analysis of Western et al. which showed that low SES populations (based on education attainment, income, and deprivation) did not benefit from digital physical activity interventions that high SES populations did [10]. For many other indicators (e.g., sexual orientation and location), it is more difficult to draw conclusions since research is sparse, highlighting the need for future studies to report these metrics [11].

## Case study: inclusiveness in a weight management trial

As one example of how inclusiveness can be examined at various stages of research, the recent Fit & Quit clinical trial conducted in the USA focused on reducing the weight gain that occurs upon quitting smoking by randomizing participants into one of three digital weight management interventions. This study included 305 participants, and 67.9% identified as women and 43.3% identified as Black/African American, with a mean age of 54.3 (standard deviation = 11.6) [52]. Researchers found that radio advertisements (i.e., on gospel, rhythm, and blues stations) were the best method for recruiting participants who identified as Black, and online recruitment strategies (e.g., Google, Facebook advertisements) were most effective for non-urban participants [15]. When examining the likelihood of proceeding to randomization, individuals identifying as Black (OR = 0.53, 95% CI = 0.33–0.84) had lower odds of proceeding from recruitment to randomization, and individuals residing in non-urban areas (OR = 8.76, 95% CI = 1.15–66.77) had higher odds of being randomized. In terms of engagement, participants identifying as White self-weighed more frequently than participants identifying with other racial groups (M (SD) = 3.0 (1.9) days/week vs. 2.3 (1.8), *p* = 0.002), with no differences observed by rurality or income. Similarly, individuals identifying as White had higher session attendance (47%) compared to those who identified with other racial groups (39% of sessions, *p* = 0.01), with no differences by rurality or income. In addition, while 12-month retention was overall high (89%), individuals who identified as Black (16.7% vs. 6.3% for White participants, *p* = 0.005) and participants with lower incomes (i.e., < $50,000) (15.7% vs. 2.4% of participants with higher incomes,* p* = 0.0002) had significantly higher attrition. Finally, all three weight management interventions were successful at reducing or eliminating post-cessation weight gain, and 46.9% of participants were successful at quitting smoking, with no race, rurality, or income-based differences (*p*s > 0.05) [52]. Thus, future applications of this research should pay particular attention to facilitating randomization, intervention engagement, and retention among participants who identify as Black as well as retention among those with lower incomes.

## Conclusions

In summary, digital health interventions have the potential to address disparities by improving reach and engagement among individuals who would not otherwise have access to in-person programs (e.g., rural populations, those with caregiving responsibilities or transportation issues, those with non-traditional work schedules, individuals with disabilities, and those with lower SES). To appropriately fulfill the promise of digital health interventions, however, researchers need to modify common research practices to improve broader knowledge of for whom, where, and why inequalities arise. Tackling these challenges, and aiming to recruit, randomize, engage, and retain a broader and more representative swath of the population in digital health trials, is crucial to the future of our field and broader support of health equity. It is our duty as researchers not to be part of the problem, but to be part of the solution.

## Data Availability

Not applicable.
